# Complex genetic interactions of novel *Suppressor of Hairless* alleles deficient in co-repressor binding

**DOI:** 10.1371/journal.pone.0193956

**Published:** 2018-03-06

**Authors:** Anette Preiss, Anja C. Nagel, Heiko Praxenthaler, Dieter Maier

**Affiliations:** Institute of Genetics (240), University of Hohenheim, Stuttgart, Germany; Oxford Brookes University, UNITED KINGDOM

## Abstract

Throughout the animal kingdom, the Notch signalling pathway allows cells to acquire diversified cell fates. Notch signals are translated into activation of Notch target genes by CSL transcription factors. In the absence of Notch signals, CSL together with co-repressors functions as a transcriptional repressor. In *Drosophila*, repression of Notch target genes involves the CSL homologue Suppressor of Hairless (Su(H)) and the Notch (N) antagonist Hairless (H) that together form a repressor complex. Guided by crystal structure, three mutations *Su(H)*^*LL*^, *Su(H)*^*LLF*^ and *Su(H)*^*LLL*^ were generated that specifically affect interactions with the repressor H, and were introduced into the endogenous *Su(H)* locus by gene engineering. In contrast to the wild type isoform, these *Su(H)* mutants are incapable of repressor complex formation. Accordingly, Notch signalling activity is dramatically elevated in the homozygotes, resembling complete absence of *H* activity. It was noted, however, that heterozygotes do not display a dominant *H* loss of function phenotype. In this work we addressed genetic interactions the three H-binding deficient *Su(H)* mutants display in combination with *H* and *N* null alleles. We included a null mutant of *Delta (Dl)*, encoding the ligand of the Notch receptor, as well as of *Su(H)* itself in our genetic analyses. *H*, *N* or *Dl* mutations cause dominant wing phenotypes that are sensitive to gene dose of the others. Moreover, *H* heterozygotes lack bristle organs and develop bristle sockets instead of shafts. The latter phenotype is suppressed by *Su(H)* null alleles but not by H-binding deficient *Su(H)* alleles which we attribute to the socket cell specific activity of Su(H). Modification of the dominant wing phenotypes of either *H*, *N* or *Dl*, however, suggested some lack of repressor activity in the *Su(H)* null allele and likewise in the H-binding deficient *Su(H)* alleles. Overall, *Su(H)* mutants are recessive perhaps reflecting self-adjusting availability of Su(H) protein.

## Introduction

The Notch signalling pathway is instrumental for a multitude of cell fate decisions during the development of higher metazoan animals. The principle outcomes of Notch activity are cells of different fate arising from a direct intercellular communication of cell neighbours [1,2]. A prime example is the process of lateral inhibition, where single cells are selected from a cell group of originally equal potential. The cells selected retain their primary fate, whereas their neighbours are directed into a secondary fate. The selection of sensory organ precursor cells giving rise to mechano-sensory bristle cells, or the refinement of a wing vein from a field of cells with provein potential, are classical examples for lateral inhibition taking place during the development of *Drosophila melanogaster* (for review: [3–5]). Failure of this process, for example as consequence of mutations in Notch signalling components, results in too many bristles or in thickened veins [6]. The opposite phenotypes, lack of bristles or veins, are observed when Notch activity is gained, and primary cell fate is completely inhibited as a consequence [3,7,8]. In addition to the process of lateral inhibition, Notch activity is also required for the formation of the dorso-ventral boundary in the wing anlagen that eventually forms the wing margin [9–11]. Accordingly, downregulation of Notch activity causes failure of wing margin formation, giving rise to wing incisions, i.e. name-giving wing ‘notches’ [3,6]. Moreover, specification of the sensory organ precursor cell’s daughters requires differential Notch activity. The outer shaft differentiates from the socket cell by a specific Notch signal. Again, loss of Notch activity may result in a double shaft, and gain of Notch activity in a double socket phenotype (for review: [12–14]).

The Notch signalling pathway, simplified, consists in *Drosophila* of the following core components (for review: [2,11,15]): two transmembrane ligands, Delta (Dl) and Serrate (Ser) presented on the signalling cell, the transmembrane receptor Notch on the signal receiving cell, plus the transcription factor Suppressor of Hairless (Su(H)) that assembles activator or repressor complexes on Notch target genes, depending on the activation status of the receptor. Once Notch is bound by Dl or Ser, it is cleaved within the membrane, and the intracellular domain—i.e. activated Notch—is released. By binding to Su(H) the Notch intracellular domain (NICD) assembles an activator complex together with Mastermind (Mam), resulting in a burst of transcriptional activity from Notch target genes (for review: [2,5,11,16]). The unligated Notch receptor remains at the membrane, leaving the cell under the rule of its antagonist named Hairless (H) (for review: [2,16,17]). H binds to Su(H), and by recruitment of general corepressors Groucho and C-terminal binding protein, it causes the silencing of Notch target genes [16–23]. Su(H) can therefore be considered a molecular switch: activating or repressing Notch target genes depending on the bound cofactors and the cellular context. Su(H) binds the two cofactors Notch and H with similar affinity at nanomolar range [24,25]. The structure of either activator or repressor complex has been determined by X-ray crystallography [24,26]. It was shown that two structural domains of Notch contact Su(H) at the surface of its beta-trefoil and C-terminal domains [24]. H instead piles into Su(H)’s C-terminal domain resulting in a large conformational change that precludes Notch binding [26]. Based on these data, we have generated three new *Su(H)* alleles by genome engineering, *Su(H)*^*LL*^, *Su(H)*^*LLF*^ and *Su(H)*^*LLL*^, specifically affecting the H-Su(H) interface [27]. Two or three amino acids required for H contact have been each mutated, resulting in a partial or complete loss of H protein binding [26]. The three alleles are homozygous lethal demonstrating the requirement of repression of Notch target genes for normal fly development. As expected by their failure of H binding, the homozygotes behave as Notch gain of function alleles indistinguishable from *H* null mutants [27]. For example, the mutant larvae display enlarged wing imaginal discs with enhanced Notch target gene expression, as well as increased lateral inhibition [27]. Only subtle allele specific differences were observed, and *Su(H)*^*LL*^ appeared to retain minimal residual activity in accordance with residual H-binding capacity [26,27]. We noted however, that the heterozygotes showed no dominant phenotypes that characterize *H* null alleles [27]. In this respect the H-binding deficient *Su(H)* alleles resemble *Su(H)* null alleles [6, 28,29].

Comprehensive genetic analyses have revealed that accurate Notch signalling relies on a well-balanced presence of its core components. In fact, among only 26 haplo-insufficient loci discovered in *Drosophila melanogaster* by extensive chromosomal deletion studies [30] three comprise the Notch core components *N*, *Dl* and *H*, whereas the majority affects ribosomal proteins. Heterozygous null mutants *N*, *Dl* or *H* display dominant phenotypes mostly affecting bristles and wings. As described above, *N* heterozygotes are characterized by wing incisions, and display thickened longitudinal veins in addition [6]. Likewise, *Dl* heterozygotes develop thickened and knotted veins, whereas *H* mutants are characterized by loss of mechano-sensory bristles or hairs and shortened and thinned wing veins [6]. Since H-binding deficient *Su(H)* alleles are expected to behave similar to *H* null mutants, we might have expected a likewise reduced bristle number or vein defects. Instead, there are no indications of a dose sensitivity for Su(H) activity. We therefore used genetic interaction studies to define gain and loss of function activities in these novel *Su(H*) mutants. Firstly, our studies confirm earlier reports on a gain of Notch activity in heterozygous *Su(H)* null alleles, which is in agreement with a loss of repressor function. Hence *Su(H)*^*attP*^ resemble *H*^*attP*^ heterozygotes in the genetic combinations similar to H-binding deficient *Su(H)* alleles. We also observed allele specific interactions of *Su(H)*^*LLF*^ and *Su(H)*^*LLL*^ with *N*^*5419*^ or *H*^*attP*^. The results suggest that *Su(H)*^*LLF*^, in addition to its failure to assemble repressor complexes with H, may also be hampered in activator complex formation. *Su(H)*^*LLL*^ however, is the one allele that resembles closest a *H* loss of function. The name giving suppression of the double socket phenotype of *H* heterozygotes, however, was only observed in combinations with the *Su(H)*^*attP*^ null allele. In contrast, H-binding deficient *Su(H)* alleles behaved more like wild type control with little influence on socket cell formation. We conclude that the dose sensitive interaction uncovered in *Su(H)*, *H* doubly heterozygotes is a result of the specific auto-regulatory activity of Su(H) during socket cell development.

## Results

### H-binding deficient *Su(H)* alleles are neither suppressors nor enhancers of *Hairless*

As indicated by its name, the *Suppressor of Hairless (Su(H))* locus was originally identified by virtue of its dosage-sensitive, antagonistic interaction with *Hairless (H)* mutants: the haplo-insufficient bristle defect of *H* loss of function alleles is suppressed in trans-heterozygous combinations with *Su(H)* loss of function alleles, whereas it is enhanced by duplications of the locus [28,29,31–33]. According to the role as Notch antagonist, mutations in *H* result in a gain of Notch activity, expected to be ameliorated by a concomitant loss of the signal transducer *Su(H)* [12,28,29,31,33]. Suppression of *H* phenotypes is restricted to bristle defects, however, and is the only dominant phenotype of *Su(H)* mutants that are otherwise fully recessive [28,29,32].

**Fig 1:**
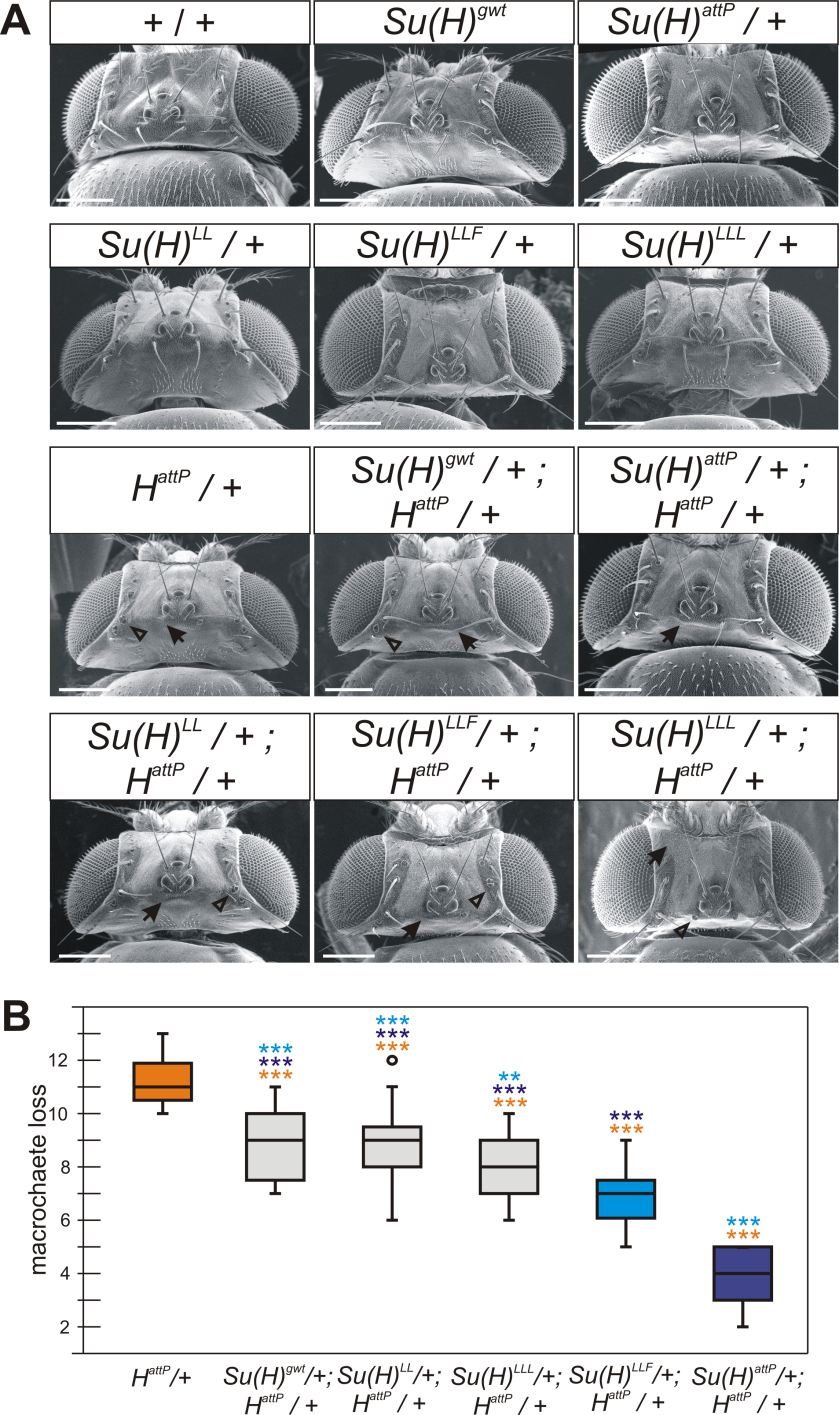
Bristle defects in the H-binding deficient *Su(H)* alleles combined with the *H*^*attP*^ null allele. *Su(H)* alleles as indicated were crossed with the null allele *H*^*attP*^, and consequences on bristle development were recorded. Flies are in Oregon R wild type background. **(A)** Scanning electron micrographs of fly heads of the given genotype. Arrows point to examples of missing bristles, and arrowheads to examples of shaft to socket transformations. Size bar, 200 μm. **(B)** Numbers of affected macrochaete (either missing or transformed to a socket) were determined in female flies (n≥20), and are represented as box plot: centre lines depict the medians, box limits indicate the 25^th^ and 75^th^ percentiles, whiskers extend 1.5 times the interquartile range, outliers are shown by dots. Statistical significance was determined by ANOVA two-tailed Tukey-Kramer approach for multiple comparisons; significant differences are indicated—color code referring to correspondingly colored box (highly significant ***, p<0.001; very significant **, p<0.01).

H-binding deficient *Su(H)* alleles cannot be recruited into H-Su(H) repressor complexes, and hence lack repressor function, which is apparent in the homozygotes as a clear increase in Notch signalling activity similar to a *H* null mutant [27]. The heterozygotes, however, do not display bristle defects like *H* mutants (Fig 1A) [27]. In fact, we have no indications for a dose dependency of these mutants as tissue hypertrophy is indistinguishable between homo- and hemizygotes, i.e. independent of whether one or two mutant gene copies are present [27]. In the heterozygous condition, bristle shafts and sockets of H-binding deficient *Su(H)* alleles, *Su(H)*^*LL*^, *Su(H)*^*LLF*^ and *Su(H)*^*LLL*^, appear wild type, indicating normal regulation of Notch signalling effecting regular cell type specification (Fig 1A). Two controls were used, wild type Oregon R flies and *Su(H)*^*gwt*^. The latter was generated by introducing a genomic wild type copy of *Su(H)* into the *Su(H)*^*attP*^ founder line as proof of principle for successful gene engineering. *Su(H)*^*gwt*^ hence has a nearly identical genetic background as the H-binding deficient *Su(H)* alleles [27].

In heterozygous *H*^*attP*^/+ females about 12 bristles are affected on average, a value that drops to about 5 in the absence of one *Su(H)* gene copy as in *Su(H)*^*attP*^ (Fig 1B). These numbers are in line with earlier observations derived from combinations with other *Su(H)* and *H* loss of function alleles [28,29,31,32]. The trans-heterozygous *Su(H)*^*gwt*^/+; *H*^*attP*^/+ flies display a slightly milder phenotype with about 9 affected bristles (Fig 1B). Similar numbers are observed in combinations with the H-binding deficient alleles that share the identical genetic background (Fig 1B). Whereas *Su(H)*^*LL*^ and *Su(H)*^*LLL*^ have little impact on the haplo-insufficient phenotype resulting from a reduction of *H* gene dose, *Su(H)*^*LLF*^ had a mild but significant suppressor effect (Fig 1B). The latter may result from structural deficits in the Su(H)^LLF^ protein uncovering some loss of Su(H) activity [26,27]. We had expected an enhancement of the *H* mutant bristle phenotype in the trans-heterozygous combinations, since in a homozygous condition the three new alleles gain Notch activity similar to *H* mutants [27]. Instead, the three alleles behaved more like the control, and only *Su(H)*^*LLF*^ behaved like a weak hypomorphic *Su(H)* allele in this context.

### Impact of H-binding deficient *Su(H)* alleles on the haplo-insufficient *H*^*attP*^ mutant wing phenotype

**Fig 2:**
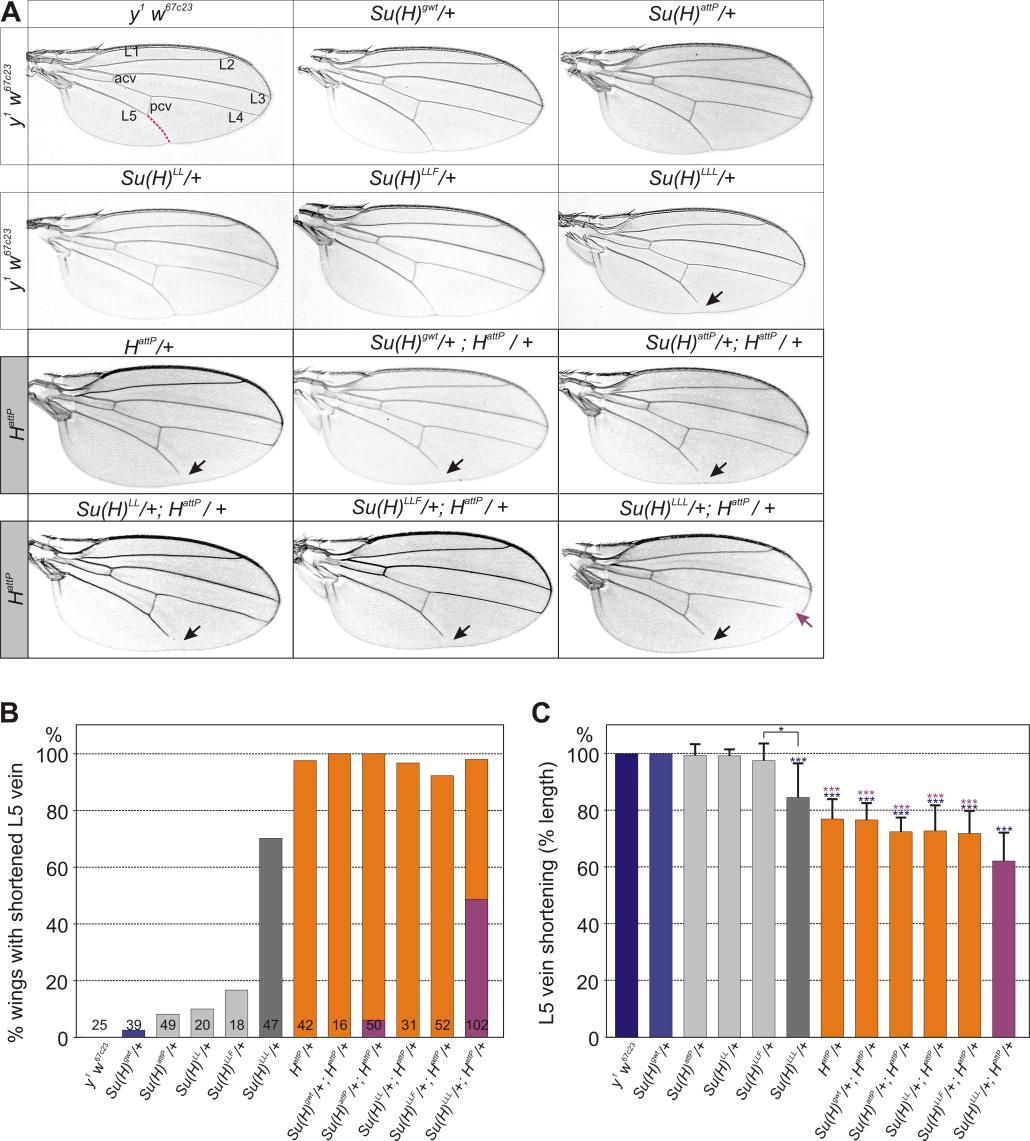
Wing phenotypes of doubly-heterozygous *H* and *Su(H)* alleles. **(A)** In the upper two rows, wings from controls and *Su(H)* mutant alleles are shown. The lower two rows show combinations with the null allele *H*^*attP*^. Typical examples of wings from female flies of the given genotype are shown. Longitudinal veins L1-L5 and anterior and posterior cross veins (acv, pcv) are labelled in the control. *H* mutant wings are characterized by a shortened L5 vein (arrow) (magenta line in control). This phenotype is likewise seen in the *Su(H)*^*LLL*^ heterozygotes with high frequency, and largely unchanged in the doubly heterozygotes of *H*^*attp*^ and any *Su(H)* mutant allele. The doubly heterozygotes of *H*^*attp*^ and *Su(H)*^*LLL*^ also show frequently gaps in L4 (purple arrow). **(B)** Quantitative analysis of wing phenotypes, summarizing percentage of wings with shortened L5 veins derived from females of the given genotype (n, total number of wings is given in each column). Columns of the doubly heterozygotes are highlighted in orange. Shortening of L4 is indicated in addition, and is shown in purple. **(C)** The length of distal L5 (indicated as a magenta line in the control wing in (A)) was measured and recorded as fraction of the expected length. Standard deviation is given (n≥16). Statistical significance was determined by ANOVA two-tailed Tukey-Kramer approach for multiple comparisons; significant differences are color labeled with blue relative to control and magenta relative to *Su(H)*^*LLL*^/+; *H*^*attP*^/+ (highly significant ***, p<0.001; significant *, p<0.05). Note little variation from control in the heterozygous *Su(H)* alleles except for *Su(H)*^*LLL*^. Again with exception of *Su(H)*^*LLL*^, the combination of *H*^*attP*^ with either *Su(H)* allele has no measurable influence on the length of L5.

Apart from the name giving bristle phenotype, *H* mutants are characterized by a distal shortening of longitudinal L5 with high, and of L4 veins with low penetrance (Fig 2A) [6]. Loss of wing veins results from increased lateral inhibition, reflecting gain of Notch activity (overview in [4,34]). By lacking H-binding we might have expected the three new *Su(H)* alleles to resemble *H* loss of function mutants. In agreement with earlier reports, shortening of the L5 vein is occasionally observed in *Su(H)* loss of function alleles [31], and likewise in *Su(H)*^*attP*^, *Su(H)*^*LL*^ and *Su(H)*^*LLF*^ heterozygotes: it occurs at very low penetrance and not significantly different from control. *Su(H)*^*LLL*^ heterozygotes, however, display shortening of L5 in a substantial fraction of wings (Fig 2A and 2B).

It has been reported that in combination with *Su(H)* loss of function alleles, the venation defects of *H* heterozygotes are enhanced [6,29,35]. We confirmed this observation: not only L5 but also L4 was affected in flies doubly heterozygous for the null mutations *Su(H)*^*attP*^ and *H*^*attP*^, albeit at low frequency (Fig 2B). Apparently in the context of wing development, a reduced repressor activity is uncovered in the *Su(H)*^*attP*^ null allele. The H-binding deficient *Su(H)* alleles, however, gave different results: when combined with *H*^*attP*^, wing phenotypes of the doubly heterozygotes were nearly unchanged regarding frequency and gap size, except for *Su(H)*^*LLL*^ (Fig 2B and 2C). Not only was the size of the L5 gap significantly increased in the *Su(H)*^*LLL*^/+; *H*^*attP*^/+ combination (Fig 2C), the L4 was affected as well in a high proportion of the wings (Fig 2B). Overall, we were surprised by these results, as we might have expected an enhancement of venation defects, affecting L4 and L2 as well also in the other combinations with *Su(H)*^*LL*^ and *Su(H)*^*LLF*^. Such an enhancement of the *H* wing venation phenotype can be seen for example in combinations with *N*^*Ax*^ mutations that gain Notch activity [36–38]. In sum, with exception of *Su(H)*^*LLL*^, enhancement of the heterozygous *H* wing phenotype was absent or rather mild, indicating that the remaining wild type copies of the *Su(H)* and *H* genes were sufficient for normal repression of Notch activity.

### Genetic interactions between H-binding deficient *Su(H)* alleles and the *Notch* loss of function allele *N*^*5419*^

**Fig 3:**
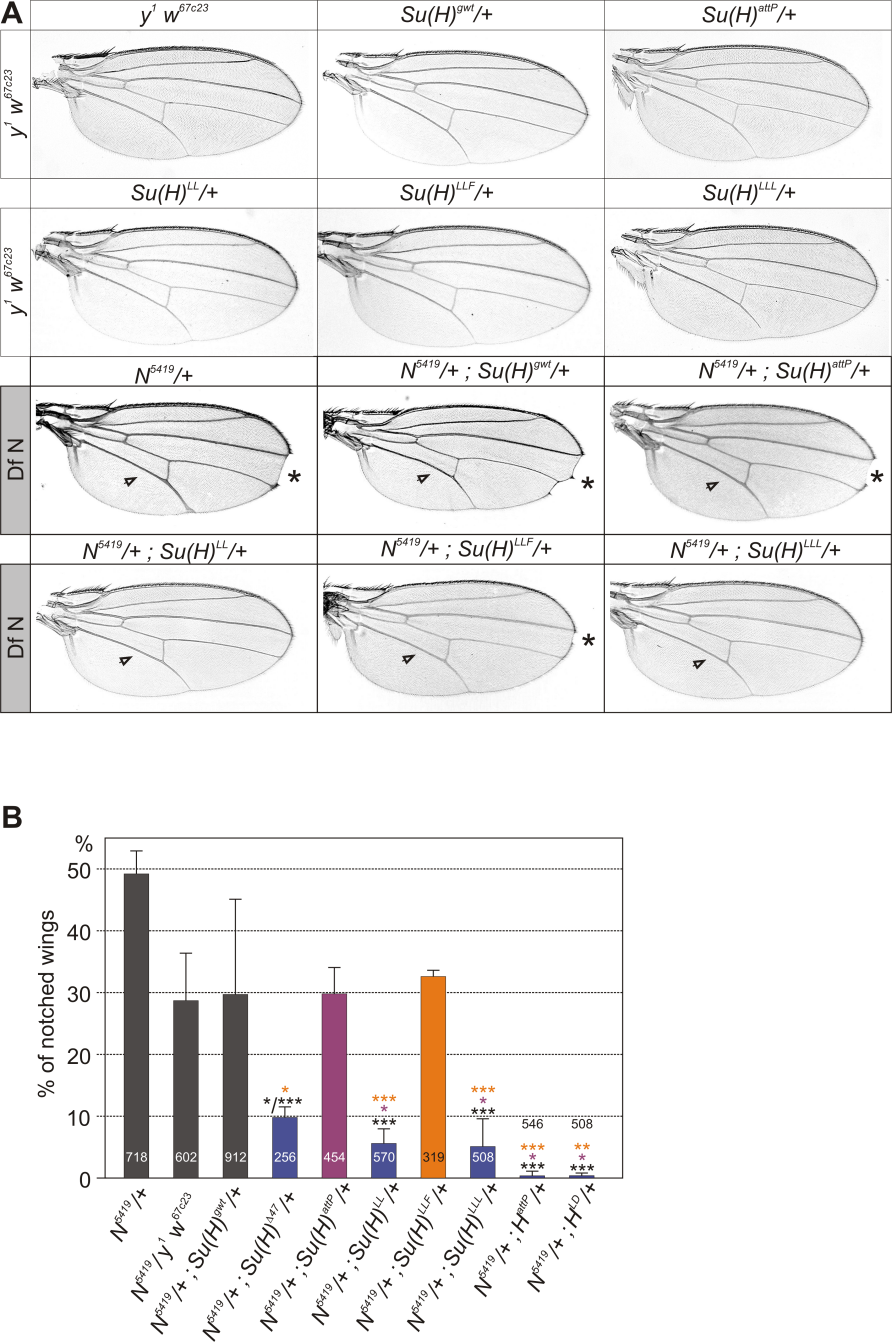
Genetic interactions of *Su(H)* alleles with the deficiency *N*^*5419*^. **(A)** Typical examples of wings from female flies of the given genotype are shown. In the upper two rows, wings from controls and *Su(H)* mutant alleles are depicted. The lower two rows show combinations with the deficiency *N*^*5419*^. *N* mutants are characterized by wing incisions (asterisk) and thickened L3 and L5 longitudinal veins (arrowhead points to L5). The doubly heterozygotes of *N*^*5419*^ and any *Su(H)* mutant allele results in an amelioration of the wing phenotypes. **(B)** Quantitative analysis summarizing percentage of notched wings derived from females of the given genotype (n, total number of wings is given in each column). Standard deviation is given from 2–4 independent experiments. Statistical significance was determined on the total by ANOVA two-tailed Tukey-Kramer approach for multiple comparisons; significant differences are color coded correspondingly (highly significant ***, p<0.001; very significant **, p<0.01; significant *, p<0.05). Note high variations in the three control crosses *N*^*5419*^/+ (derived from *N*^*5419*^/FM7c x Oregon R), *N*^*5419*^/*y*^*1*^ *w*^*67c23*^, and *N*^*5419*^/+; *Su(H)*^*gwt*^/+. The two null alleles *Su(H)*^*Δ47*^ and *Su(H)*^*attP*^ show drastically different genetic interactions–the former rescuing *N* wing phenotypes, the latter behaving rather like a wild type allele. Moreover, *N*^*5419*^/+; *Su(H)*^*LLF*^/+ does not differ from the controls.

*Notch* mutants were originally picked up by their characteristic notched wing phenotype observed in heterozygous females [6,39] (Fig 3). This phenotype is exquisitely sensitive to genetic background [6,39–41], and can be completely rescued by reducing the gene dose of *Delta* or *Hairless* [42–44]. We reasoned that H-binding deficient *Su(H)* alleles might behave similar to *H* mutants in this context, as we expected them to gain Notch activity, whereas *Su(H)* null alleles were expected to enhance loss of Notch activity. To our surprise we found that the deficiency *Su(H)*^*Δ47*^ rescued wing notching of *N*^*5419*^ heterozygous null mutants very well, whereas the null allele *Su(H)*^*attP*^ did not (Fig 3). We noted a high degree of variations, however, between independent experiments as well as between lines with formally identical genetic background. Similarly, high variability was observed amongst the three controls *N*^*5419*^/+ (derived from *N*^*5419*^/FM7c x Oregon R), *N*^*5419*^/*y*^*1*^ *w*^*67c23*^, and *N*^*5419*^/+; *Su(H)*^*gwt*^/+ (Fig 3) in line with the background sensitivity of *N* phenotypes [6,41–44]. In fact, *Su(H)*^*attP*^ did not vary significantly from these controls, and neither did *Su(H)*^*LLF*^. As noted in the context of bristle development, the *Su(H)*^*LLF*^ mutant appears a hypomorphic mutant in the *N*/+ mutant background as well (Fig 3). The two other H-binding deficient *Su(H)* alleles, *Su(H)*^*LL*^ and *Su(H)*^*LLL*^, however, gained Notch activity and rescued the wing defects very well, thereby resembling the *H*^*attP*^ null mutant and the Su(H)-binding deficient *H*^*LD*^ allele (Fig 3B). Notably, *H* alleles had the greatest potential in rescuing Notch defects (Fig 3B). We cannot decide, however, whether these results reflect meaningful differences in biological activity or are due to genetic background [41].

### Genetic interactions between H-binding deficient *Su(H)* alleles and the *Delta* loss of function allele *Dl*^*B2*^

**Fig 4:**
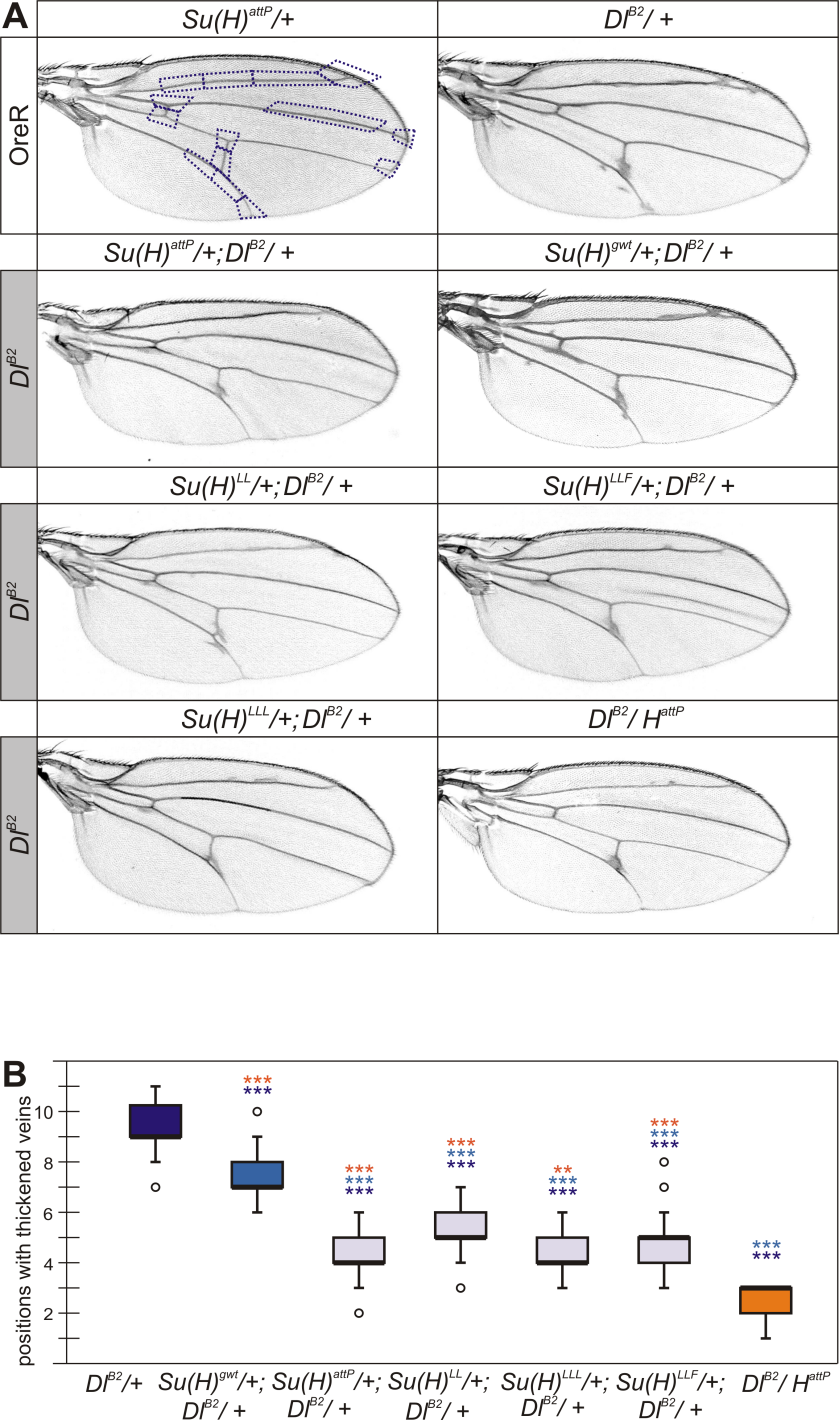
Genetic interactions of *Su(H)* alleles with the null mutant *Dl*^*B2*^. **(A)** Typical examples of wings from female flies of the given genotype are shown. Controls are in the upper row: *Su(H)*^*attP*^ heterozygous flies have wings with wild type appearance, whereas *Dl*^*B2*^ mutants typically display vein thickening and knotting along the longitudinal veins L2-L5 as well as at the anterior and posterior cross veins. Areas typically affected in *Dl*^*B2*^ are highlighted by blue dotted boxes in the control wing, and were quantified in (B). These are ameliorated in the combination with any *Su(H)* allele. **(B)** Quantitative analysis of genetic interactions. Female wings of the given genotype (n≥15) were monitored for thickening at fourteen positions as highlighted in (A). Vein thickening at any of these positions was valued 1, wild type was valued 0. The Y-axis represents the values of positions with phenotypic aberrations per wing. A box plot is shown with centre lines representing the median, box limits indicate the 25^th^ and 75^th^ percentiles, whiskers extend 1.5 times the interquartile range and outliers are represented by dots. Statistical significance was determined by ANOVA two-tailed Tukey-Kramer approach for multiple comparisons; significant differences are color labeled correspondingly (highly significant ***, p<0.001; very significant **, p<0.01).

*Delta* heterozygous mutant flies display a thickening and knotting of veins, notably along the longitudinal veins as well as the cross veins. Moreover, veins frequently run out in a delta at the wing margin, which is the name giving phenotype [6,45] (Fig 4). Interestingly, this phenotype is ameliorated by both gain and loss of Notch activity. Doubly heterozygous *Dl /H* flies show nearly wild type wings [42,44,46], i.e. both dominant phenotypes are compensated for, which can be explained by a loss of Notch activity in the absence of *Dl* and a gain of Notch activity in the absence of *H*. Both *Dl* and *N* wing phenotypes are ameliorated in doubly heterozygous *N*/+; *Dl*/+ flies as well [42]. This phenotype has been accounted for by balancing the gene dose of ligand and receptor, or more likely by cis-inhibition [3,47]. We have combined the null allele *Dl*^*B2*^ with the H-binding deficient *Su(H)* alleles, and noted an amelioration of vein thickening (Fig 4). In this context, the three H-binding deficient *Su(H)* alleles were indistinguishable from the null allele *Su(H)*^*attP*^. The rescue, however, was clearly less effective than that obtained with the *H*^*attP*^ null allele (Fig 4B). Unexpectedly, we also observed a significantly weakened phenotype in the *Su(H)*^*gwt*^/+; *Dl*^*B2*^/+ combination compared to *Dl*^*B2*^/+ alone (Fig 4B). The effect was clearly weaker than with the other *Su(H)* alleles or with *H*^*attP*^, however (Fig 4B). Whether it results from genetic background or whether the *Su(H)*^*gwt*^ allele lacks some Su(H) activity cannot be distinguished at the moment [41].

## Discussion

In this work, we have studied the genetic interactions between H-binding deficient *Su(H)* alleles and mutations affecting several Notch pathway components. The results are complex, perhaps reflecting the dual role of Su(H) in Notch signal transduction as activator and repressor alike [11,16,17]. In the activator complex together with NICD and Mam, Su(H) acts as a transcriptional activator of Notch target genes, whereas it acts as a repressor when in complex with H and corepressors. Accordingly, *Su(H)* loss of function mutants display mixed phenotypes, resulting from inhibition of Notch target gene activation as well as relief of repression, depending on the context [48–51]. In our experiments, *Su(H)/+* null mutants gain Notch activity resembling *H*/+ mutants. For example, the wing phenotypes of both, *Dl* and *N* heterozygotes are ameliorated by lowering the *Su(H)* gene dose (Figs 3 and 4). We might have expected the opposite result, i.e. phenotypic enhancement, as Su(H) protein is required to transmit Notch signalling activity. Our results, however, are in agreement with earlier observations of the rescue of *Dl*^*9P39*^ and enhancement of *N*^*Ax-E2*^ dominant wing phenotypes by the *Su(H)*^*AR9*^ hypomorph [35]. Similarly, in the doubly heterozygous combination of *Su(H)* and *H*, the *H* wing venation phenotype was enhanced rather than suppressed [28,29,32]. Moreover, the loss of bristle organs was increased, in contrast to the double-socket phenotype (see below) [32]. These results highlight the repressor function of Su(H): limited amounts of Su(H) appear to limit repressor complex rather than activator complex availability. H-binding deficient *Su(H)* alleles fail at repressor complex formation. Accordingly, they resemble *Su(H)* null alleles in heterozygosis. This is generally observed in all the combinations tested, with exception of *Su(H)*^*LLF*^ and *Su(H)*^*LLL*^ that display allele specific interactions when combined with *N*^*5419*^ and *H*^*attP*^, respectively. *Su(H)*^*LLF*^ stood out because it suppressed *H* bristle loss significantly stronger than either *Su(H)*^*LL*^ or *Su(H)*^*LLL*^, behaving as a subtle *Su(H)* hypomorph in this context (Fig 1). In fact, in combination with *N*^*5419*^ heterozygotes it was undistinguishable from the *Su(H)*^*attP*^ null allele (Fig 3). We conclude that *Su(H)*^*LLF*^, in addition to its incompetence for repressor complex formation, has a reduced ability to form Notch activation complexes perhaps as a consequence of structural deficits [26]. In contrast, *Su(H)*^*LLL*^ is the one allele that resembles mostly the *H*^*attP*^ null mutant also in the heterozygous condition, judged by the wing venation defects this allele displays on its own and in combination with *H* (Fig 2). Other than that, H-binding deficient *Su(H)* alleles are fully recessive, unlike *H*. Apparently, one wild type copy of *Su(H)* is sufficient for normal Notch pathway repression, and the mutant *Su(H)* gene copy does not increase Notch output in any detectable way.

The core components of Notch signalling in *Drosophila*, the ligand Delta, the receptor Notch and the co-repressor Hairless are dose sensitive, i.e. mutations are haplo-insufficient and cause dominant phenotypes [6]. Curiously, mutations in the two central players, the co-activator Mastermind (Mam) and the transcription factor Su(H), are recessive, suggesting that these two factors are not limiting in the process of Notch signal transduction [6,29,30]. Mam together with NICD and Su(H) assemble the activator complex [24]. As recruitment of Mam strictly relies on the presence of NICD, it has no effect on Notch target gene expression on its own [11,24]. Hence it can be hold available in excess without influencing signalling output. The picture is quite different for Su(H): in the absence of NICD, Su(H) may engage in other transcriptional complexes impacting gene expression [11,16,52]. The classical example is repressor complex formation together with H and co-repressors [16,17]. Hence, *Su(H)* copy numbers are expected to influence the outcome of Notch signalling activity- and still, *Su(H)* mutations are recessive. This puzzle may be solved by regulated availability of Su(H) protein. Our recent work provides evidence that Su(H) protein is stabilised by interactions with transcription-regulator complexes involving H or NICD [27]. Accordingly, Su(H) protein was detected at very low levels in cells lacking H, and likewise was H-binding deficient Su(H) protein in wild type cells. Moreover, NICD was sufficient to stabilise both wild type and mutant Su(H) protein [27]. Even if Su(H) protein were to be expressed in excess, it may not be available at promoters in the nucleus, because Su(H) protein appears to have a short half life if not bound to either H or NICD [27]. Consequently, Su(H) protein levels within a cell are self correcting, strictly depending on H and/or NICD levels, explaining the lack of phenotypes in heterozygous *Su(H)* mutants.

*Su(H)* alleles have been identified originally by their dominant suppression of the bristle defects normally observed in heterozygous *H* mutants [28,29,32] (Fig 1). *H* heterozygotes either completely lack the entire bristle organ or just the bristle shaft, which is instead transformed into a bristle socket [12,53,54]. The resultant double sockets are observed in place of normal bristle organs [44,54–56]. Loss of complete bristle organs in *H* mutants originates from increased Notch signalling within the proneural field, preventing selection of the bristle founder cell [12,54]. The double socket phenotype reflects the role of Notch in asymmetric cell type specification within the bristle lineage: the sensory organ precursor cell divides asymmetrically into a cell pair of outer and inner fate (pIIa and pIIb), with pIIa eventually giving rise to shaft (trichogen) and socket (tormogen) cells [12,14,54,57]. Gain of Notch activity prevents the asymmetry, and drives for example trichogen into tormogen fate, resulting in the typical double socket phenotype observed in heterozygous *H* mutant flies [44, 54,57,58]. Interestingly, suppression of *H* phenotypes by *Su(H)* is restricted to the double sockets, i.e. the decrease of Su(H) levels improves trichogen formation in this sensitized genetic background [29,32].

Su(H) plays an active role within the tormogen, whereas the trichogen forms by default repression of Notch signals [12,51,59,60]. Su(H) is required within the socket cell, as a pIIa cell lacking Su(H) gives rise to shaft cells only [33,61]. Su(H) protein accumulates to high levels within the tormogen by means of an autoregulatory element (ASE) driving Su(H) expression in response to Notch activity [32,59–62]. One of Su(H) targets is *Sox15*, and together the two proteins entail socket cell differentiation as well as normal electrophysiology of the bristle organ [59,60]. Moreover, they inhibit the transcription of *shaven*, a gene required for shaft cell differentiation [60,63]. *shaven* is expressed in the late sensory organ precursor cell and its daughters, but later on expression is only maintained in cells protected from Notch signal [63]. Protection from Notch signals is the prime role of the two proteins Numb and Hairless that antagonize Notch activity [12,14,17,57,61]. By enabling *shaven* expression, they set a bias for the shaft fate [60,63]. Accordingly, the double socket phenotype of *H* is strongly enhanced by *shaven* mutants [53], and that of *numb* is suppressed by *Su(H)* [61].

In the heterozygous *H* mutant, commitment to the trichogen fate has become unstable, since Notch activity cannot be completely abolished. Spurious Notch signals may switch on *Su(H)* expression via the ASE, resulting in the activation of *Sox15* and the repression of *shaven*, eventually driving socket cell differentiation. In this *H*/+ context, *Su(H)* appears dose sensitive: The lowered *Su(H)* dose may be insufficient for spurious Notch activity to trigger ASE activation, and *shaven* expression—i.e. trichogen bias—is maintained. H-binding deficient *Su(H)* alleles have little influence on the trichogen-tormogen fate selection (Fig 1). In these mutants, the NICD binding sites are untouched [24,26]. Activator complex assembly is expected to occur normally [26], as is the response to Notch receptor activation within the presumptive tormogen. These *Su(H)* alleles should be similar to wild type with regard to the activation of Notch target genes including ASE regulation, which is in agreement with our observations. Overall, the heterozygous condition of *Su(H)* is fully recessive except for the sensitized *H*/+ genetic background regarding the tormogen-trichogen cell lineage. If any, a slight gain of Notch activity is uncovered in *Su(H)* heterozygotes, reflecting the important role of Su(H) in the default repression of Notch target genes.

## Materials and methods

Flies were maintained on standard fly food at 18°C; crosses were raised at 25°C. The following stocks were used: Oregon R (BL5), *y*^*1*^ *w*^*67c23*^ (BL6599), *H*^*attP*^ / TM6B, *H*^*LD*^/TM6B [44], *Su(H)*^*gwt*^, *Su(H)*^*LL*^ / CyO, *Su(H)*^*LLF*^ / CyO, *Su(H)*^*LLL*^ / CyO, [27], *Dl*^*B2*^ / TM6C *Sb* (BL5602) [64], Df(1)N-5419 / FM7c (BL6894) [65], *Su(H)*^*Δ47*^/CyO [66]. Further information on fly strains is found in Flybase (flybase.org) and the Bloomington Stock Centre (http://flystocks.bio.indiana.edu/). Standard genetics were applied for the re/combination of fly stocks. Where applicable, genotypes were confirmed by PCR plus diagnostic restriction digests where applicable. Mutant phenotypes were documented as outlined before [44]. Adult wings from female flies were dehydrated in ethanol and mounted in Euparal (Roth, Karlsruhe, Germany). Pictures were taken with an ES120 camera (Optronics, Goleta CA, USA) mounted to a Zeiss Axiophot (Carl Zeiss AG, Jena, Germany) using *Pixera Viewfinder* software, version 2.0. Scanning electron micrographs were captured with a table-top scanning electron microscope (Neoscope JCM-5000; Nikon, Tokyo, Japan) of uncoated animals. Pictures were assembled using *Photo Paint* and *Corel Draw* software. *Image J* was used for measurements of vein length. Statistical significance was determined by ANOVA using a two-tailed Tukey-Kramer approach for multiple comparisons (highly significant ***, p<0.001; very significant **, p<0.01; significant *, p<0.05; not significant ns, p>0.05). Box blots were compiled with the online plotting tool *BoxPlotR*. The raw data are contained in S1 Table.

## Supporting information

S1 Table Raw data. The file contains the raw data used for the statistical analysis presented in Figs 1–4.
(XLS)
